# Quality of life, voiding and sexual function of penile cancer patients: DaPeCa‐10—a cross‐sectional questionnaire survey

**DOI:** 10.1002/bco2.159

**Published:** 2022-05-17

**Authors:** Jakob Kristian Jakobsen, Christian Møbjerg Sørensen, Kim Predbjørn Krarup, Jørgen Bjerggaard Jensen

**Affiliations:** ^1^ Department of Urology Aarhus University Hospital Aarhus Denmark; ^2^ Department of Urology Rigshospitalet, Copenhagen University Hospital Copenhagen Denmark; ^3^ Department of Clinical Medicine Aarhus University Aarhus Denmark

**Keywords:** penile cancer, quality of life, sexual life, voiding symptoms

## Abstract

**Objectives:**

To assess prevalence of voiding and sexual symptoms and quality of life in penile cancer patients.

**Methods:**

From 1 January 2013 to 31 December 2015, we approached three separate groups of Danish penile cancer patients and asked them to complete a face‐validated questionnaire at diagnosis (Group 1), after 1 year (Group 2) and after 2 years (Group 3). We analysed symptom prevalence and bother and quality of life items and explored differences between groups.

**Results:**

In total, we analysed 157 questionnaires. The response rates at diagnosis, after 1 year and after 2 years were 29%, 46% and 30%. The pad use (*p* = 0.001) and occurrence of nocturia twice a night or more (*p* = 0.006) was significantly decreasing 2 years after treatment. There was an increasing trend in sexual thoughts and importance of sexuality from 1 to 2 years after treatment, but the proportion of patients reporting a frequency of orgasm at more than once in the past 6 months was significantly decreasing after treatment (*p* = 0.03). Likewise, the trend for erectile dysfunction worsened after treatment with 49% of patients reporting an erection never sufficient for intercourse at diagnosis increasing to 62% after 1 year and 69% after 2 years. We observed trends towards lower self‐esteem with increasingly mutilating treatment.

**Conclusion:**

Pad use, nocturia and frequency of orgasm were significantly reduced after penile cancer treatment. We observed trends towards lower self‐esteem with increasingly mutilating treatment and increase in erectile dysfunction after treatment.

## 1 INTRODUCTION

Among a multitude of morphs in penile cancer, especially tumours at the external urethral orifice, larger glans involving or eroding lesions and tumours presenting with the clinical picture of complete phimosis may all cause voiding symptoms. Following resection involving the external urethral orifice and distal urethra, glansectomy, partial penectomy and total penectomy surgeons will perform a reconstruction of the urethral orifice, which in turn may lead to a new perception of voiding and varying challenges with urine stream control and need for urinary aids.

Many treatments of penile cancer may affect penile sensibility, reduce sensible areas, introduce deformity and reduce length. Some of these changes may inflict sexual challenges on patients, and in some cases, the sum of changes caused by disease and treatment may affect quality of life.^1^, ^2^, ^3^, ^4^, ^5^

Even if a cancer is perceived life‐changing and body‐image‐altering, a number of human psychological defence mechanisms and survival instincts prevail in the dominating narrative of many patients, who mobilize remarkable coping strategies and thrive against difficult odds.^3^, ^6^, ^7^, ^8^

Few studies have focused on differences at different time points in aspects of quality of life, voiding symptoms and sexual function after treatment for penile cancer.^9^ Such data may be useful in the planning of cancer rehabilitation and might guide the focus and themes of the clinical follow‐up.

In this study we assess quality of life, voiding and sexual function related to three different points in time: at diagnosis, after 1 year and after 2 years. We assess outcomes related to type of treatment, and we compare Danish penile cancer patients with healthy Scandinavian men. This study is among the first to focus on quality of life and sexuality in penile cancer at different time points in the clinical trajectory.

## 2 METHODS

We conducted a questionnaire survey among Danish penile cancer patients from 1 January 2013 to 31 December 2015 in connection with inpatient treatment and outpatient follow‐up. The study was prospectively designed as a two‐centre, three‐time point cross‐sectional questionnaire‐based survey in penile cancer patients. All patients were treated and followed up at the only two Danish urology departments treating penile cancer with curative intent. Questionnaires were handed out, introduced and collected as a part of everyday clinical practice by health professionals involved in the clinical trajectory. There was no dedicated study specific organization.

We approached patients if they were newly diagnosed (Group 1) if they attended a 1‐year follow‐up visit in our outpatient clinics (Group 2) or if they attended a 2‐year follow‐up visit (Group 3) during the study period. Patients registered to have accepted participation but failing to return the questionnaire received an identical questionnaire, a pre‐stamped envelope and a reminding letter per mail 1 month after inclusion. We excluded patients who failed to respond to first and second invitation to participate.

To assess response rates, we retrospectively extracted the patterns of referral to the two specialized centres treating penile cancer with a curative intent in Denmark from the Danish National Penile Cancer Database.

### 2.1 Questionnaire

The questionnaire queried patients on a wide range of issues with focus on demographic data, psychological aspects of life, voiding symptoms and sexual experiences and symptoms. We developed the study specific questionnaire from a combination of validated questionnaires and a series of purpose‐built single questions. We translated into Danish and adapted for penile cancer patients the validated Scandinavian Prostate Cancer Group Study Number 4 (SPCG‐4) Questionnaire.^10^ The SPCG‐4 Questionnaire is partly based on other validated tools and two psychometric tests such as Spielberger's Trait measure from the State–Trait Anxiety Inventory^11^ and the Centre for Epidemiological Studies Measure of Depression.^12^ The psychological symptoms (anxiety, depressed mood), sense of well‐being and quality of life were assessed on 7‐point visual digital scales. We assessed the resulting penile cancer questionnaire for face validity in a pilot project with six patients. Because the pilot project only led to minor non‐significant changes to the original questionnaire, we included these six patients in the study cohort. We considered responders sexually active if they had engaged in any form of sexual stimulation or intercourse 6 months prior to responding to the questionnaire.

### 2.2 Comparison

We compared score prevalence between groups of penile cancer patients according to time of completion of the questionnaire and according to the type of treatment received.

### 2.3 Statistics

We ensured that data were discrete and independent by only considering one response per patient. In 27 patients responding more than once, we only considered their latest response. We compared age between groups by ANOVA and differences between groups of other demographic and clinical variables by nonparametric tests. For statistical analysis we used Stata Statistical Software: Release 13, TX: StataCorp. We considered a *p*‐value of 0.05 or less statistically significant.

### 2.4 Sexological counselling

Coinciding with study initiation, we decided to offer all penile cancer patients free in‐hospital post‐operative sexological counselling by a specially trained nurse as a standard of care. Thus, all study participants were offered post‐operative sexological counselling.

## 3 RESULTS

**FIGURE 1:**
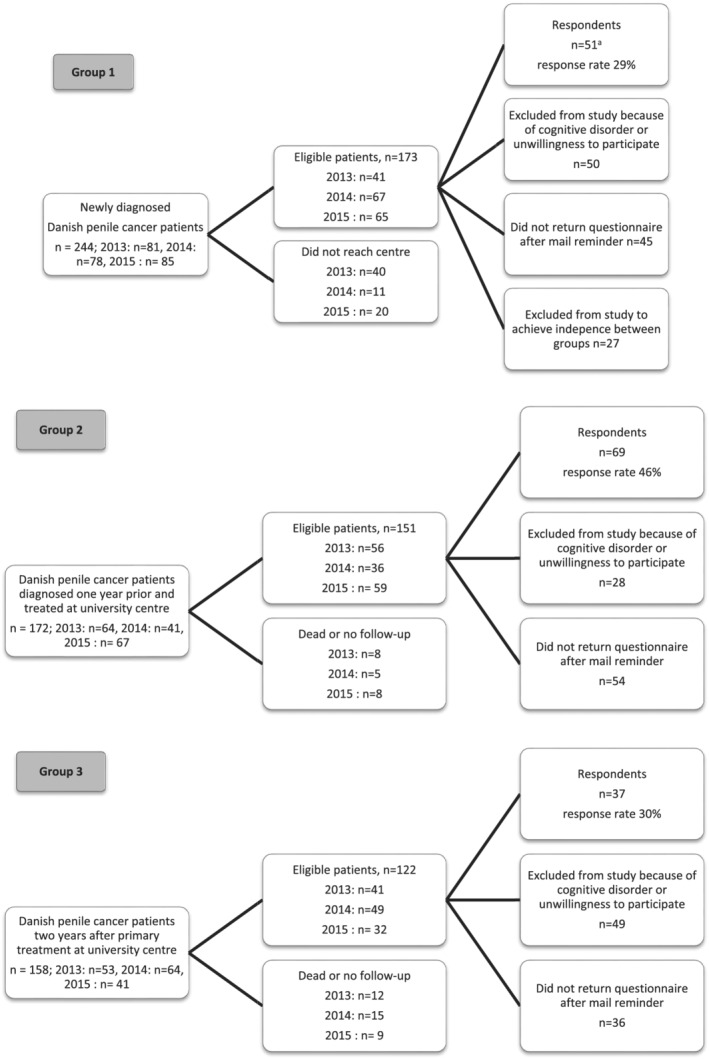
Patient inclusion and data collection. ^a^Six of 51 patients participated in pilot study for questionnaire face validity

The response rate of the questionnaires in Groups 1, 2 and 3 were 51/173 (29%), 69/151 (46%) and 37/122 (30%) (Figure 1). In total, we analysed 157 questionnaires from individual patients. Only two responses were collected following a reminding letter. The mean age of responders at the time of responding was 70.3 years in Group 1, 67.2 years in Group 2 and 66.4 years in Group 3 (Table 1). We recruited 145 patients (92%) in Centre 1.

**TABLE 1:** Patient characteristics for three groups of Danish penile cancer patients responding to a validated quality of life questionnaire at diagnosis (Group 1), after 1 year (Group 2) and after 2 years or more (Group 3)

Variable	Group 1 (*n* = 51)	Group 2 (*n* = 69)	Group 3 *(n* = 37)
Age, years			
Mean	70.3	67.4	66.4
Median	71.3	69.0	68.3
Range	43.6–91.7	46.9–92.5	41.2–87.2
Body mass index (BMI) kg/m^2^			
Mean	28.8	27.4	26.5
Median	27.8	26.7	26.1
Range	23.1–40.8	19.6–41.7	19.7–42.9
Marital status, *n* (%)			
Married or cohabiting	38 (74)	50 (73)	24 (65)
Living alone without a partner	8 (16)	12 (17)	7 (19)
Living alone, but has a partner	4 (8)	4 (6)	4 (11)
Widower	1 (2)	3 (4)	2 (5)
Educational level			
Compulsory school only	29 (57)	44 (64)	25 (68)
Upper secondary school	8 (16)	16 (23)	7 (19)
Higher education	14 (27)	9 (13)	5 (13)
Source of income			
Employed	16 (31)	16 (23)	13 (35)
Unemployed/looking for a job	0	2 (3)	0
Sickness pension/sickness benefit	2 (4)	5 (7)	3 (8)
Retired/pension benefit	33 (65)	46 (67)	21 (57)
Performance status^a^			
0	32 (63)	45 (65)	25 (68)
1	16 (31)	20 (29)	9 (24)
2	2 (4)	4 (6)	2 (5)
3	1 (2)	0	0
4	0	0	1 (3)
Type of treatment			
Local resection and/or laser	‐	35 (51)	18 (49)
Partial penectomy	‐	20 (29)	14 (38)
Total Penectomy	‐	14 (20)	5 (13)
Treatment Centre 1/2	39/12	69/0	37/0

^a^ Perfomance status according to the Eastern Cooperative Oncology Group: 0 = fully active, able to carry on all pre‐disease performance without restriction, 1 = restricted in physically strenuous activity but ambulatory and able to carry out work of a light or sedentary nature, for example, light house work and office work, 2 = ambulatory and capable of all self‐care but unable to carry out any work activities; up and about more than 50% of waking hours, 3 = capable of only limited self‐care; confined to bed or chair more than 50% of waking hours, 4 = completely disabled; cannot carry on any self‐care; totally confined to bed or chair.

### 3.1 Symptoms and distress associated with urinary functions in penile cancer patient groups

At the current group sizes, we found few differences in symptom prevalence or bother scores between penile cancer patient groups 1, 2 and 3 (Table 2). There was a significant difference in the self‐reported occurrence of nocturia 24/51 (47%) in Group 1, 33/68 (49%) in Group 2 and 8/36 (22%) in Group 3, *p* = 0.006. Treatment significantly reduced the need for pad use from 14/50 (28%) in Group 1 to 5/68 (7%) and 2/36 (6%) in Groups 2 and 3, *p* = 0.001. There was a trend towards increased voiding frequency 6/51 (12%) in Group 1 to 13/67 (19%) and 12/36 (33%) in Groups 2 and 3, *p* = 0.15.

**TABLE 2:** Symptoms and distress associated with sexual and urinary functions for three groups of Danish penile cancer patients responding to a validated questionnaire at diagnosis (Group 1), after 1 year (Group 2) and after 2 years or more (Group 3)

Variable	Outcome definition	Group 1 (*n* = 51)	Group 2 (*n* = 69)	Group 3 (*n* = 37)	*p*‐value^a^
Desire					
Sexual thoughts	Occurrence more than once a month	40/52 (78%)	47/68 (69%)	28/35 (80%)	0.40
Sexuality					
Importance of sexuality	Moderate or great importance	18/51 (35%)	20/68 (29%)	15/36 (42%)	0.76
Sexuality part of one's manhood	Yes	35/51 (69%)	48/68 (71%)	27/35 (77%)	0.17
Ability to sexually satisfy partner	Seldom or never	32/47 (68%)	44/65 (68%)	22/32 (69%)	0.38
Distress from decreased sexual ability	Moderate to great distress	18/51 (35%)	27/66 (41%)	13/33 (39%)	0.90
Penile stiffness					
Erectile function	Never sufficient for intercourse	25/51 (49%)	42/68 (62%)	24/35 (69%)	0.30
At awakening	Never sufficient for intercourse	29/51 (57%)	45/68 (66%)	25/37 (68%)	0.25
Intercourse					
Frequency of intercourse	More than once in past 6 months	19/51 (37%)	23/68 (34%)	11/35 (31%)	0.44
Distress from decreased frequency	Moderate to great distress	10/51 (20%)	18/67 (27%)	9/32 (28%)	0.81
Orgasm					
Frequency of orgasm	More than once in past 6 months	29/50 (58%)	30/68 (44%)	11/33 (33%)	0.03^b^
Urinary emptying symptoms					
Weak stream	More than half of all occasions	17/51 (33%)	15/66 (23%)	11/35 (31%)	0.29
Feeling of incomplete emptying	More than half of all occasions	4/51 (8%)	15/68 (22%)	9/36 (25%)	0.39
Frequent voiding, interval <2 h	More than half of all occasions	6/51 (12%)	13/67 (19%)	12/36 (33%)	0.15
Difficulty to start voiding	More than half of all occasions	4/51 (8%)	4/67 (6%)	4/36 (11%)	0.12
Urinary storing symptoms					
Nocturia	Occurrence twice a night or more	24/51 (47%)	33/68 (49%)	8/36 (22%)	0.006^b^
Urgency	Occurrence once a day or more	2/51 (4%)	5/68 (7%)	2/36 (6%)	0.56
Distress from voiding problems	Moderate or great distress	6/51 (12%)	4/69 (6%)	5/35 (14%)	0.90
Urinary leakage					
Urinary leakage in daytime	Occurrence once a week or more	7/51 (14%)	6/68 (9%)	2/36 (6%)	0.33
Urinary leakage in daytime	Occurrence once a day or more	0/51	2/68 (3%)	1/36 (3%)	0.33
Regular dependence on some form of protective aid against urinary leakage	Pad, yes	14/50 (28%)	5/68 (7%)	2/36 (6%)	0.001^b^
	Diaper, yes	1/48 (2%)	2/65 (3%)	2/32 (6%)	0.34
	Urisheath, yes	1/48 (2%)	2/65 (3%)	2/32 (6%)	0.34
	Other aid, yes	0/47	1/65 (2%)	0/30	0.87

*Notes*: Data are *n*/*N* (%) where *N* is the total number of patients responding to a given question. For each question, some men did not respond.

^a^ Non‐parametric test for trend across groups.

^b^ Significant result.

### 3.2 Symptoms and distress associated with sexual functions in penile cancer patient groups

Trends towards increasing occurrence of sexual thoughts from first (69%) to second year (80%) after diagnosis (*p* = 0.40) and an increase in attributing moderate or great importance to sexuality from first (29%) to second year (42%) after diagnosis (*p* = 0.76) did not reach statistical significance. This was also true for the trend of increasing erectile dysfunction 25/51 (49%) in Group 1 to 42/68 (62%) and 25/37 (69%) in Groups 2 and 3, *p* = 0.30. The frequency of orgasm was reported with a significant decline in the number of responders having more than one orgasm 6 months prior to responding 29/50 (58%) in Group 1 to 30/68 (44%) and 11/33 (33%) in Groups 2 and 3, *p* = 0.03. The fraction of responders reporting that sexuality is a part of one's manhood displayed a trend in increasing score with time from diagnosis 35/51 (69%) in Group 1 to 48/68 (71%) and 27/35 (77%) in Groups 2 and 3, *p* = 0.17.

### 3.3 Comparison of psychological symptoms between penile cancer groups

Table 3 summarizes comparison of penile cancer patient responses to psychological questionnaire items. At the current study size, we observed no significant psychometrical score differences between groups. Trends towards a declining fraction of patients scoring ‘high’ in perceived quality of life, sense of meaningfulness, physical coping, physical well‐being and self‐esteem during the preceding 6 months were not significant (*p* = 0.61, *p* = 0.57, *p* = 0.14, *p* = 0.40 and *p* = 0.17).

**TABLE 3:** Occurrence and intensity of psychological symptoms for Danish penile cancer patients responding to a validated questionnaire

Variable	Group 1 (*n* = 51)	Group 2 (*n* = 69)	Group 3 (*n* = 37)	*p*‐value^a^
Quality of life (high)	31/51 (61%)	37/67 (55%)	15/34 (44%)	0.61
Sense of meaningfulness (high)	30/51 (59%)	40/67 (60%)	18/35 (51%)	0.57
Physical coping (high)	32/51 (63%)	39/67 (58%)	16/35 (46%)	0.14
Depressed mood (moderate or high)	20/51 (39%)	37/69 (54%)	17/36 (47%)	0.36
Anxiety (moderate or high)	25/51 (49%)	39/69 (57%)	15/36 (42%)	0.51
Psychological well‐being (high)	25/51 (49%)	36/69 (52%)	17/36 (46%)	0.61
Physical well‐being (high)	32/51 (63%)	38/68 (56%)	18/36 (50%)	0.40
Self‐esteem (high)	30/51 (59%)	38/68 (56%)	15/37 (41%)	0.17

*Notes*: Data are *n*/*N* (%) where N is the total number of patients responding to a given question. For each question, some men did not respond.

^a^ Non‐parametric test for trend across groups.

### 3.4 Comparison of psychological symptoms between treated penile cancer groups

Table 4 summarizes comparison of treated penile cancer patient responses according to treatment type. At the current study size, we observed no significant score differences between patients treated with distinct types of treatment. Pooling data for patients from Group 2 and 3 and allocating patients into broader treatment categories ‘local treatment’ and ‘penectomy’ did not change this finding. Trends towards less self‐esteem in patients subjected to more mutilating treatment were seen in both Groups 2 and 3 but did not reach statistical significance.

**TABLE 4:** Occurrence and intensity of symptoms for treated Danish penile cancer patients from Groups 2 and 3 analysed per type of treatment

Variable	Group 2 *n* = 69			*p*‐value^a^	Group 3 *n* = 37			*p*‐value^a^
	Local resection *n* = 35	Partial penectomy *n* = 20	Total penectomy *n* = 14		Local resection *n* = 18	Partial penectomy n = 14	Total penectomy *n* = 5	
Sense of meaningfulness (high)	21/33 (67%)	11/20 (55%)	8/14 (57%)	0.52	10/17 (59%)	6/14 (43%)	2/4 (50%)	0.44
Physical coping (high)	21/33 (67%)	11/20 (55%)	7/14 (50%)	0.61	9/17 (53%)	6/14 (43%)	1/4 (25%)	0.19
Depressed mood (moderate or high)	16/35 (46%)	14/20 (70%)	7/14 (50%)	0.79	9/18 (50%)	6/14 (43%)	2/4 (50%)	0.78
Anxiety (moderate or high)	19/35 (54%)	14/20 (70%)	6/14 (43%)	0.66	7/18 (39%)	7/14 (50%)	1/4 (25%)	0.60
Psychological well‐being (high)	18/35 (51%)	9/20 (45%)	9/14 (64%)	0.61	9/18 (50%)	5/13 (38%)	3/5 (60%)	0.76
Physical well‐being (high)	21/35 (60%)	10/19 (53%)	7/14 (50%)	0.96	9/18 (50%)	7/14 (50%)	2/4 (50%)	0.46
Self‐esteem (high)	21/35 (60%)	9/19 (47%)	8/14 (57%)	0.96	10/18 (56%)	4/14 (29%)	1/5 (20%)	0.14

*Notes*: Data are *n*/*N* (%) where N is the total number of patients responding to a given question. For each question, some men did not respond.

^a^ Non‐parametric test for trend across groups.

### 3.5 Sexological counselling

Four out of 157 (2.5%) men accepted the free offer of in‐hospital post‐operative sexological counselling during the study period either as a solo counselling session or as a couple counselling session with a partner. Further two patients received self‐paid counselling in an out‐of‐hospital setting, one by a private practice clinical sexologist and one by a psychologist.

## 4 DISCUSSION

This study is among the first to investigate symptoms and distress associated with sexual and urinary functions and psychological domains in penile cancer patients with data from different time points in the clinical trajectory. In this large questionnaire survey, we found several interesting trends and some noteworthy findings that enable us to increase the level of detail in preoperative patient information and counselling. During the study, we learned important aspects about collecting patient reported outcome measures from penile cancer patients and about offering sexological counselling to penile cancer patients that will allow us to pursue these factors in future studies and daily practice.

When asking penile cancer patients at diagnosis, 29/50 (58%) reported more than one orgasm, and 19/51 (37%) reported more than intercourse in the 6 months preceding the diagnosis. In other words, many penile cancer patients are still sexually active even in the last months before diagnosis. Penile cancer treatment, however, significantly reduces the proportion of responders having more than one orgasm in the preceding 6 months 1 year after treatment 30/68 (44%) and 2 years after treatment11/33 (33%), *p* = 0.03. This is in line with previous studies on the subject.^1^, ^4^, ^5^, ^9^ Although illness and treatment affect sexuality, some patients continue an active sex life after surgery with reports of more than one intercourse in the preceding 6 months from 23/68 (34%) 1 year after treatment and 11/35 (31%) 2 years after treatment.

Oozing and bleeding from cancerous lesions and occasional urinary dribbling because phimosis may cause penile cancer patients to use pads, which was the case for 22% of patients at diagnosis. After 1 and 2 years, this figure declined dramatically to 7% and 6%, *p* = 0.006. As treatment removes oozing lesions and resolves phimosis, this finding is not surprising but nonetheless an important aspect in preoperative reassurance of patients.

The implementation of several items from the SPCG‐4 questionnaire in our penile cancer questionnaire allows us to compare our findings to a large control cohort of cancer‐free Scandinavian men from the SPCG‐4 study.

We find a significant difference between the proportion of responders scoring the importance of sexuality as moderate or great with 53/155 (34%) of penile cancer patients versus 118/204 (58%) in the SPCG‐4 control group. There was a significant difference between the proportion of responders reporting a frequency of intercourse at more than once in past 6 months 53/154 (34%) of penile cancer responses versus 106/203 (52%) control group responses, *p* = 0.02 A significantly larger proportion of penile cancer patient responses scored anxiety as moderate or high compared with the SPCG‐4 control group responses: 79/156 (51%) versus 68/208 (33%), *p* = 0.02. There was no difference in scores for depressed mood, well‐being, quality of life and sense of meaningfulness.^13^ Nocturia more than twice a night was reported by 40% of penile cancer patients and 42% of the slightly older controls, *p* = 0.81.

In summary, as has been reported by previous penile cancer studies, we also found in the current study that both disease and treatment had negative effects on sexual aspects of life, such as reported frequency of intercourse, reported frequency of orgasm and judged importance of sexuality, which were all scored significantly lower by penile cancer patients than SPCG‐4 cancer‐free controls.^1^, ^2^, ^5^, ^13^

During the study period, only four (2.5%) of patients accepted our free offer of in‐hospital post‐operative sexological counselling. Further two patients reported self‐paid counselling in an out‐of‐hospital setting. This was surprising to us because we expected a larger group of men to accept the free offer of in‐hospital post‐operative sexological counselling. The hesitance of men to accept this offer might have to do with the setting of a busy and sometimes hectic outpatient clinic environment in which they were invited. This environment could discourage patients to engage in discussions of existential, sexual and emotional aspects of the disease and treatment. Most patients accepting the offer did so at a later time points after diagnosis and treatment, which might indirectly point to the fact that men might not be ready to engage in discussion and counselling of the sexological aspect of the disease at this early time point.

### 4.1 Limitations and strengths

This study has a number of limitations. Due to small sample size and low response rate, we cannot claim to have established insights about penile cancer patients in general. A major limitation that is inherent in all penile cancer studies is the small cohort size. Our response rates at 29%, 46% and 30% in the three approached groups of the questionnaire survey remained low despite reminder letters mailed to non‐responders. In a Dutch quality of life and sexuality questionnaire study among penile cancer patients, they reached a response rate above 60 with a questionnaire with fewer items than in the current study.^5^ We tried to run this study along with normal outpatient activities in two busy university clinic settings without dedicated study staff. Some of our approached and non‐responding patients may have been discouraged to participate due to the substantial number of items in our questionnaire. One obvious way to approach this challenge may be to markedly reduce the number of items. Future attempts to collect patient reported outcome measures should focus on fewer items and either be integrated in obligatory electronic logistic procedures connected to the outpatient clinic routine or have dedicated data collection staff (or both) to ease participation and increase response rates. Nevertheless, penile cancer is a rare disease, and in fact, this is one of the larger series published on the subject. Our cohort represents national data with responders from both treating centres in Denmark that limits selection bias. However, we do see some indirect signs of selection bias in the dataset in our counterintuitive age distribution between Groups 1, 2 and 3, where one would expect the age to increase with time after diagnosis, the distribution of responders in our study is opposite, with more young patients participating in the group 2 years after treatment. We interpret this as a selection bias of the health professionals enrolling patients for Groups 2 and 3 in the study being more likely to invite younger patients than older patients. Only few patients were recruited from Centre 2. Because we have no questionnaire from non‐responders, we have no way to compare base line characteristics from patient responses, but we are able to indirectly draw some conclusions from our knowledge of the penile cancer cohort as a whole. Our responders have a higher mean age at 70.3 years, 67.4 years and 66.4 years compared with our general penile patient cohort at 66.1 years,^14^ which indicates that a larger proportion of the younger patients declined to participate in the current questionnaire study. We speculate that this might have skewered our data in the direction of less dramatic differences between scores, because it is our experience from a previous single patient interview study^3^ and from our daily practice that younger patients seems to worry more about consequences of disease and treatment than older patients. If we look at other baseline parameters and compare our responders to our penile cancer cohort as a whole, we find no difference in cohabitation, education level and source of income.^15^

Nearly 60% of responding penile cancer patients are sexually active at diagnosis. Pad use, nocturia and frequency of orgasm were significantly reduced after penile cancer treatment. We observed trends towards lower self‐esteem with increasingly mutilating treatment and decline in erectile function after treatment. Only few patients accepted our free offer of in‐hospital post‐operative sexological counselling. We need to improve recruitment logistics and increase response rates in future studies to further explore the patient perspective in this rare disease.

## CONFLICT OF INTEREST

The authors report no conflicts of interest. The authors alone are responsible for the content and writing of the paper.

## ETHICS STATEMENT

We received approval of the research protocol by the Institutional Reviewer Board. We conducted the study in accordance with Danish law and rules and regulations from regional authorities. The study received permission from the Danish Data Protection Agency, file number 1‐16‐02‐95‐1‐3. All patients consented to participate.

## AUTHOR CONTRIBUTIONS

Study concept and design: Jørgen B. Jensen and Jakob K. Jakobsen. Data Collection: Kim P. Krarup, Christian M. Sørensen, Jørgen B. Jensen and Jakob K. Jakobsen. Analysis and interpretation of data: Christian M. Sørensen and Jakob K. Jakobsen. Drafting of the Manuscript: Jakob K. Jakobsen. Critical revision of the manuscript for important intellectual content: Kim P. Krarup, Christian M. Sørensen, Jørgen B. Jensen and Jakob K. Jakobsen. Statistical Analysis: Jakob K. Jakobsen. Study Supervision: Jørgen B. Jensen.

## Supporting information


**Data S1.** Supporting Information

## References

[1] Maddineni SB , Lau MM , Sangar VK . Identifying the needs of penile cancer sufferers: A systematic review of the quality of life, psychosexual and psychosocial literature in penile cancer. BMC Urol. 2009;9:8. 10.1186/1471-2490-9-8 PMID: 19664235 19664235 PMC2731105

[2] Drager DL , Milerski S , Sievert KD , Hakenberg OW . Psychosocial effects in patients with penile cancer; a systematic review/Psychosoziale Auswirkungen bei Patienten mit Peniskarzinom; Ein systematischer Uberblick Urologe. Ausgabe a. 2018;57(4):444–52. 10.1007/s00120-018-0603-9 29476193

[3] Mortensen GL , Jakobsen JK . Patient perspectives on quality of life after penile cancer. Dan Med J. 2013;60(7):A4655. PMID: 23809966 23809966

[4] Windahl T , Skeppner E , Andersson SO , Windahl T , Skeppner E , Andersson SO , et al. Sexual function and satisfaction in men after laser treatment for penile carcinoma. J Urol. 2004;172(2):648–51. 10.1097/01.ju.0000132891.68094.87 PMID: 15247753 15247753

[5] Kieffer JM , Djajadiningrat RS , van Muilekom EA , Graafland NM , Horenblas S , Aaronson NK . Quality of life for patients treated for penile cancer. J Urol. 2014;192(4):1105–10. 10.1016/j.juro.2014.04.014 PMID: 24747092 24747092

[6] Borgi M , Collacchi B , Ortona E , Cirulli F . Stress and coping in women with breast cancer: Unravelling the mechanisms to improve resilience. Neurosci Biobehav Rev. 2020;119:406–21. 10.1016/j.neubiorev.2020.10.011. Epub 2020 Oct 18 PMID: 33086128 33086128

[7] Vaughan E , Koczwara B , Kemp E , Freytag C , Tan W , Beatty L . Exploring emotion regulation as a mediator of the relationship between resilience and distress in cancer. Psychooncology. 2019;28(7):1506–12. 10.1002/pon.5107 Epub 2019 Jun 6. PMID: 31087804 31087804

[8] Manne SL , Myers‐Virtue S , Kashy D , et al. Resilience, positive coping, and quality of life among women newly diagnosed with gynecological cancers. Cancer Nurs. 2015;38(5):375–82. 10.1097/NCC.0000000000000215 PMID: 25521911; PMCID: PMC4470889 25521911 PMC4470889

[9] Opjordsmoen S , Fosså SD . Quality of life in patients treated for penile cancer. A follow‐up study. Br J Urol. 1994;74(5):652–7. 10.1111/j.1464-410x.1994.tb09200.x PMID: 7827818 7827818

[10] Steineck G , Helgesen F , Adolfsson J , Dickman PW , Johansson JE , Norlén BJ , et al. Scandinavian prostatic Cancer group study number 4. Quality of life after radical prostatectomy or watchful waiting. N Engl J Med. 2002;347(11):790–6. 10.1056/NEJMoa021483 PMID: 12226149 12226149

[11] Spielberger CD , Gorsuch RL , Lushene PR , Vagg PR , Jacobs GA . Manual for the state‐trait anxiety inventory Palo Alto, Calif: Consulting Psychologists Press; 1983.

[12] Radloff LS . The CFS‐D scale: A self‐report depression scale for research in the general population. Appl Psych Meas. 1994;1:385–401. 10.1177/014662167700100306

[13] Johansson E , Steineck G , Holmberg L , et al. Long‐term quality‐of‐life outcomes after radical prostatectomy or watchful waiting: the Scandinavian Prostate Cancer Group‐4 randomised trial. Lancet Oncol. 2011;12(9):891–9. 10.1016/S1470-2045(11)70162-0 Epub 2011 Aug 5 21821474

[14] Jakobsen JK , Kortsen D , Jensen JB . DaPeCa5 ‐ Obesity at the time of diagnosis does not predict poor cancer‐specific survival in patients with penile squamous cell carcinoma ‐ a Danish national study. Scand J Urol. 2020;54(5):420–5. 10.1080/21681805.2020.1803399 32772767

[15] Baekhøj Kortsen D , Predbjørn Krarup K , Jakobsen JK . DaPeCa‐9 – Cohabitation and socio‐economic conditions predict penile cancer‐specific survival in a national clinical study from Denmark. Scand J Urol. 2021;55(6):486–90. 10.1080/21681805.2021.1879928 33554693

