# Supplementary material for: Quality of life, voiding and sexual function of penile cancer patients: DaPeCa‐10—a cross‐sectional questionnaire survey
Source: BJUI Compass. 2022 May 17;3(5):354–62. doi: 10.1002/bco2.159 (PMC9349586; doi:10.1002/bco2.159)
Supplement: Supplementary file 1 — Data S1. Supporting Information [file BCO2-3-354-s001.docx]

**Penile Cancer Questionnaire**

**Health and Quality of Life**

This questionnaire contains questions about urinary tract, sexual and quality of life. In connection with penile cancer one may experience symptoms and challenges of different kinds. We want to find out what problems you as a patient have been affected by, and how you experience them. With greater knowledge we hope to be able to minimize these inconveniences in the future.

In this questionnaire, we will ask questions about your disease, treatment and how you experience the role of a patient. The questions are most often formulated so that you answer by putting a cross in the box for the answer that best corresponds with your experience. Some of the questions may be answered by choosing several alternatives that are listed after the question.

We greatly appreciate your participation and it will be valuable if you try to answer all questions.

It is easy to skip a question or perhaps even an entire page when you

are answering the questionnaire so feel free to take a second look at

the questions. You are also given the chance to write your own

comments.

Some of the questions about, among other things, sex and sexuality,

may seem to be a bit too intimate. It is important, however, to ensure

that correct information can be given to future patients even about

these matters.

The questionnaire is by law a secret document. The results will be communicated in a way that individuals cannot be recognized.

If you need help or if you have any questions, please feel free to ask the staff at the department

thanks in advance

many kind regards

project physicians

[JKJ] and [JB]

**INTRODUCTORY QUESTIONS**

**Questions about yourself**

Put a cross in the box for the alternative that best corresponds with

your situation or experience – **only one alternative**. Write your

answer on the dotted line.

1) What year were you born? 19…………

2) Which date is today?..............

3) What is your current body weight in kilogrammes?.........kg

4) What is your current height without shoes ? ……………cm

5) Are you currently:

 Married or sharing a household

 Living alone without a partner

 Living alone but have a partner

 Widower

6) Are you currently:

 Employed

 Retired

 On long term sick leave

 Retired due to a health condition

7) What is your level of education? Check your highest level of

education:

 Compulsory schooling or equivalent

 High school or equivalent

 University or college

Feel free to write your own comments about yourself

.................................................................................................................

8) When did you first discover signs of the change you now know is penile cancer (date and year)……………………………………..

9) When did you first consult a doctor with the change you now know is penile cancer (date and year)……………………………..

10) Have you been to a dermatologist with the change you now know is penile cancer

 Yes, once

 Yes, more than once

 No, never

 I do not know

11) Can your foreskin be brought back so that the entire head of the penis can be seen?

 Yes

 No, but it was like that earlier

 No, it was never like that

 I do not know

12) Can you see your penis without using a mirror

 Yes

 No, it is not possible as the belly is in the way

 No, it is not possible as my eyesight is poor

 No, it is not possible due to other reasons……………….

**Sexually Transmitted Diseases**

13) Have you ever had genital warts (condyloma) on the penis?

 Yes

 No

 I do not know

14) Have you ever had Chlamydia, Gonorrhea or Syphilis ?

 Yes

 No

 I do not know

15) Have you ever had Herpes on the penis?

 Yes

 No

 I do not know

16) Have you ever had a penile yeast infection?

 Yes

 No

 I do not know

17) Have you ever had an HIV test performed?

 Yes, once

 Yes, more than once

 No

 I do not know

**Tobacco and Alcohol**

18) Do you smoke daily (at least 1 cigarette / cigar or pipe bowl per day)?

 Yes

If yes, for how many years have you been smoking daily?...........yrs.

 No, but I have smoked daily in the past

 No, I have never smoked daily

19) When you think back on the last year, how many items of alcohol have you been drinking on average per week? (Write number of items, write 0 if you have not been drinking at least 1 item per week)

……………beers per week

……………glasses of wine per week

……………units (2 centiliters) of liquor (distilled spirits) per week

.................................................................................................................

.................................................................................................................

.................................................................................................................

.................................................................................................................

.................................................................................................................

**QUALITY OF LIFE**

**Questions about your quality of life during the past 6 months**

20) ***During the past 6 months,*** how has your quality of life been?

*Put a circle around the number that corresponds best with your evaluation*

1---------------2-------------3-------------4---------------5-------------6-----------7

No quality of life The best possible quality of life

21) ***During the past 6 months,*** has your life felt meaningful?

*Put a circle around the number that corresponds best with your evaluation*

1---------------2-------------3-------------4---------------5-------------6-----------7

Never All of the time

22) ***During the past 6 months***, how well have you been able to manage

physically?

*Put a circle around the number that corresponds best with your evaluation*

1-------------2-------------3---------------4----------------5-----------6-----------7

Only with great difficulty Extremely well

23) ***During the past 6 months,*** have you felt dejected or depressed?

*Put a circle around the number that corresponds best with your evaluation*

1-------------2-------------3---------------4----------------5-----------6-----------7

Never All of the time

24) ***During the past 6 months,*** have you felt worried or suffered from

anxiety?

*Put a circle around the number that corresponds best with your evaluation*

1-------------2-------------3---------------4----------------5-----------6-----------7

Never All of the time

25) ***During the past 6 months,*** how has your psychological state of well

being been?

*Put a circle around the number that corresponds best with your evaluation*

1-------------2-------------3---------------4----------------5-----------6-----------7

No sense of well being Best possible sense of well being

26) ***During the past 6 months,*** how has your physical health been?

*Put a circle around the number that corresponds best with your evaluation*

1-------------2-------------3---------------4----------------5-----------6-----------7

Worst possible physical health Best possible health

27) ***During the past 6 months,*** how has your self esteem been?

*Put a circle around the number that corresponds best with your evaluation*

1-------------2-------------3---------------4----------------5-----------6-----------7

No self esteem Best possible self esteem

**DEJECTION AND WORRY**

**Questions about how you have felt during the past 6 months**

28) ***During the past 6 months*,** have you had difficulty in sleeping at night?

 No, never

 Yes, at least once during the past 6 months

 Yes, at least once a month

 Yes, at least once a week

 Yes, at least 3 times a week

 Yes, every night

29) ***During the past 6 months***, have you awakened some time during the

night with a feeling of worry, anxiety or discomfort?

 No, never

 Yes, at least once during the past 6 months

 Yes, at least once a month

 Yes, at least once a week

 Yes, at least 3 times a week

 Yes, every night

30) ***During the past 6 months***, have you experienced periods of

intense unrest, anxiety or panic (for example with heart

palpitation, breathing distress or dizziness)?

 No, never

 Yes, at least once during the past 6 months

 Yes, at least once a month

 Yes, at least once a week

 Yes, at least 3 times a week

 Yes, every day

31) ***During the past 6 months,*** have you experienced a feeling that

something terrible is happening?

 No, never

 Yes, at least once during the past 6 months

 Yes, at least once a month

 Yes, at least once a week

 Yes, at least 3 times a week

 Yes, every day

32) ***During the past 6 months,*** have you taken sleeping pills?

 No, never

 Yes, at least once during the past 6 months

 Yes, at least once a month

 Yes, at least once a week

 Yes, at least 3 times a week

 Yes, every night

33) ***During the past 6 months,*** have you taken medicine (sedatives) to calm

you down?

 No, never

 Yes, at least once during the past 6 months

 Yes, at least once a month

 Yes, at least once a week

 Yes, at least 3 times per week

 Yes, every day

34) ***During the past 6 months,*** have you taken anti-depressive

medicine (medication for anxiety and depression)?

 No, never

 Yes, every day

35) Are you depressed?

 No, I am not depressed

 Yes, I am a little depressed

 Yes, I am moderately depressed

 Yes, I am very depressed

Feel free to write your own comments about how you feel

…………………………………………………………………………

…………………………………………………………………………

…………………………………………………………………………

…………………………………………………………………………

…………………………………………………………………………

**URINARY TRACT**

**Questions about how you urinate**

Put a cross in the box for the alternative that best matches your

experience – **only one alternative**

36) ***During the past 6 months,*** how often have you had a feeling that

your bladder has not been emptied even though you have

urinated?

 Never

 On **fewer** than half of the occasions when I have urinated

 On **more** than half of the occasions when I have urinated

 Always

37) ***During the past 6 months,*** how often have you needed to urinate

within two hours?

 Never

 On **fewer** than half of the occasions I have had to urinate

within two hours

 On **fewer** than half of the occasions I have had to urinate

within two hours

 Always

38) ***During the past 6 months,*** how often have you noticed that your

urine stream was weak when you urinated?

 Never

 My urine stream has been weak on **fewer** than half of the occasions

 My urine stream has been weak on **more** than half of the occasions

 Always

39) ***During the past 6 months,*** how often have you had to exert

pressure in order to begin urinating?

 Never

 I have had to exert pressure on **fewer** than half of the occasions

 I have had to exert pressure on **more** than half of the occasions

 Always

40) ***During the past 6 months,*** how often have you gotten up to

urinate during a **typical night**?

 Never

 Approximately once

 Approximately twice

 Approximately 3 times

 Approximately 4 times

 Approximately 5 or more times

41) ***During the past 6 months,*** have you had a sudden feeling

(a bladder urgency) that you needed to urinate immediately?

 Never

 At least once during the past 6 months

 Yes, at least once a month

 Yes, at least once a week

 Yes, at least 3 times a week

 Yes, at least once a day

 Yes, at least twice a day

42) ***During the past 6 months,*** have you leaked urine during **the day**?

 *Not relevant,* I do not leak urine during the day

 Yes, at least once during the past 6 months

 Yes, at least once a month

 Yes, at least once a, I leak urine

 Yes, at least 3 times a week

 Yes, at least once a day

 Yes, at least twice a day

43) ***During the past 6 months,*** have you used one or more of the

following aids to keep from leaking urine into your clothes?

(*Answer all of the following questions)*

 *Not relevant*, I do not leak urine

A) Incontinence pads  No  Yes

B) Diapers  No  Yes

C) Uridome  No  Yes

D) Other protection  No  Yes

44) ***During the past 6 months,*** have your problems with urination led

to your avoiding doing something that really interests you (for

example a leisure time activity or accepting an invitation)?

 *Not relevant,* I do not have any difficulty urinating

 No

 Yes, at least once during the past 6 months

 Yes, at least once a month

 Yes, at least once a week

 Yes, at least 3 times a day

 Yes, at least once a day

 Yes, at least twice a day

45) If you, for the rest of your life, have to live with the same **overall**

problems with urination, how would you feel?

 *Not relevant,* I do not have any difficulty urinating

 This would not affect me at all

 This would affect me a little

 This would affect me moderately

 This would affect me very much

Feel free to write your own comments about how you urinate and about incontinence products………………………………………………………………………….………………………………………………………………………………………………………………………………………………………………………………………………………………………………………………………………………………………………..

**SEX LIFE**

Sex life is an important part of life for many people while for

others it is not so important. Sexual functioning can be affected by

penile disease. Sex is not simply sexual intercourse but can also

involve close physical contact, other erotic experiences or one’s

own personal satisfaction. In the following questions, sexual activity must be understood in a broad sense.

We want once again to remind you that the questionnaire is by law a secret document. The results will be communicated in a way that individuals cannot be recognized.

The first part of the questions are about your sex life throughout your life.

Put a cross in the box for the alternative that best matches your

experience – **only one alternative**

46) How many sex partners did you have throughout your life?

 Approximately 0-1

 Approximately 2-4

 Approximately 5-9

 At least 10 or more

47) How many sex partners did you have before you turned 20 years old?

 0

 1

 Approximately 2-3

 At least 4 or more

48) How old were you at your first intercourse?

 16 years old or younger

 17 -19 years old

 20 years old or older

49) How many times have you had sex with prostitutes?

 0

 1

 Approximately 2-3

 At least 4 or more

50) How many male sex partners did you have throughout your life?

 0

 1

 Approximately 2-3

 At least 4 or more

51) How often during your life have you used a condom during intercourse

 Never

 In less than half of my intercourses

 In more than half of my intercourses

 Always

The following questions are about your sex life during the past 6 months.

Put a cross in the box for the alternative that best matches your

experience – **only one alternative**

52) ***During the past 6 months,*** have you had thoughts about

sex?

 No, never

 Approximately once during the past 6 months

 Approximately once a month

 Approximately once a week

 Approximately 3 times a week

 Approximately once a day

53) ***During the past 6 months***, how stiff has your penis been during

sexual activity?

 *Not relevant, I have not been sexually active*

 My penis has never been sufficiently stiff for intercourse

 My penis has been sufficiently stiff for intercourse on **fewer**

than half of the occasions

 My penis has been sufficiently stiff for intercourse on **more**

than half of the occasions

 My penis has always been sufficiently stiff for intercourse

54) ***During the past 6 months,*** if you have had an erection in the

morning, how stiff was your penis?

 *Not relevant*, I have not had a morning erection

 My penis has never been sufficiently stiff for intercourse

 My penis has been sufficiently stiff for intercourse on **fewer**

than half of the occasions

 My penis has been sufficiently stiff for intercourse on **more**

than half of the occasions

 My penis has always been sufficiently stiff for intercourse

55) ***During the past 6 months,*** how often during sexual activity have

you reached orgasm?

 *Not relevant*, I have not been sexually active during the past 6

months

 Never

 I have reached orgasm during **fewer** than half of the

occasions

 I have reached orgasm during **more** than half of the

occasions

 Always

56) **Before** you received the diagnosis of penile cancer, had you

experienced diminished erection?

 Yes

 No

57) Is your penis shorter now than when you were 30 years old?

 Yes

 No

58) **If** your penis is shorter now compared with when you were 30

years old and if this condition were to continue for the rest of your

life, what do you think of that?

 *Not relevant*, my penis is not shorter

 This would not affect me at all

 This would affect me slightly

 This would affect me moderately

 This would affect me very much

59) ***During the past 6 months,*** how often have you had sexual

intercourse?

 Never

 Approximately once during the past 6 months

 Approximately 1 to 2 times a month

 Approximately 3 to 4 times a month

 Approximately 5 or more times a month

60) ***During the past 6 months,*** if you have had sexual intercourse **less**

**often** than in the past and if this situation were to continue for the

rest of your life, what do you think of that?

 *Not relevant*, I have not had intercourse less often than in the past

 This would not affect me at all

 This would affect me slightly

 This would affect me moderately

 This would affect me very much

61) ***During the past 6 months,*** have you had a partner with whom you have been sexually active?

 No

 Yes

62) ***During the past 6 months,*** have you felt that you could sexually satisfy your partner?

 *Not relevant*, I do not have a partner

 No, I never satisfy my partner

 Yes, I satisfy my partner **fewer** than half of the times I try

 Yes, I satisfy my partner **more** than half of the times I try

 Yes, I can always satisfy my partner

63) How important is sex for you at present?

 Not at all important

 Of slight importance

 Moderately important

 Very important

64) Do you believe that sexuality is part of your being a man (manhood)?

 No

 Yes

65) ***During the past 6 months***, have you avoided sexual activity because of fear that you would fail?

 No, not true at all

 Yes, this is somewhat true

 Yes, this is largely true

 Yes, this is completely true

66) If ***during the past 6 months***, your sexual capacity has been **worse**

in comparison with what it previously was and if it could be

expected to remain that way for the rest of your life, what would

you think about that?

 *Not relevant*, my sexual capacity has not become worse

 That would not affect me at all

 That would affect me a little

 That would affect me moderately

 That would affect me very much

Feel free to write your own comments on sexuality

.………………………………..................................................................

...................................................................................................................

...................................................................................................................

.................................................................…..……………………………

…………………………………………………………………………

…….……………………………………………….………..…………

…………………………………………………………………………

…………………………………………………………………………

…………………………………………………………………………

…………………………………………………………………………

**Questions regarding your health in general**

***Have you had one or more of the following illnesses during the past year?***

67) High blood pressure? Yes  No 

Do you take any medicine for this? Yes  No 

Which?..........................................................................................

.

68) Congestive heart failure? Yes  No 

Do you take any medicine for this? Yes  No 

69) Angina? Yes  No 

Do you take any medicine for this? Yes  No 

Which?..........................................................................................

70) Heart attack? Yes  No 

Do you take any medicine for this? Yes  No 

Which?..........................................................................................

71) Blood clot or bleeding in the brain or Yes  No the consequences of this?

Do you take any medicine for this? Yes  No 

Which?......................................................................................

72) Any other neurological illness? Yes  No 

Do you take any medicine for this? Yes  No 

Which?..........................................................................................

.

73) Lung problems of any kind? Yes  No 

Do you take any medicine for this? Yes  No 

Which?..........................................................................................

.

74) Ulcers or other stomach problems? Yes  No 

Do you take any medicine for this? Yes  No Which?..........................................................................................

75) Diabetes? Yes  No 

Do you take any medicine for this? Yes  No 

Which?..........................................................................................

76) Psychological problems or illness? Yes  No 

Do you take any medicine for this? Yes  No 

Which?..........................................................................................

77) Long lasting pain? Yes  No 

Do you take medicine for this? Yes  No 

Which?..........................................................................................

.

78) Any other cancer than penile cancer? Yes  No 

Do you take medicine for this? Yes  No 

Which?..........................................................................................

.

79) Do you take blood thinning medicine

(anticoagulant medicine)? Yes  No 

If yes, which?...............................................................................

80) Do you have any other illness or medication? Yes  No

Do you take medicine for this? No  Yes 

Which?....................................................................................................

.................................................................................................................

.................................................................................................................

.................................................................................................................

.................................................................................................................

................................................................................................................

[.]

You have contributed your time and your experience to advance penile

cancer research.

We express our thanks to you for your participation!

We want once again to remind you that the questionnaire is by law a secret document.

The results will be transmitted in such a way that no individual can be identified.

It is easy to mistakenly skip over a question or even a whole page. Feel free to go through the entire questionnaire one last time.

**If** you have missed an entire section in the questionnaire, may we contact you in order get a supplementary answer?

 Yes

 No

Thank you very much for your participation, the questionnaire can be returned to the staff at the department.
